# Aqueous synthesis of tin- and indium-doped WO_3_ films *via* evaporation-driven deposition and their electrochromic properties†

**DOI:** 10.1039/d1ra00125f

**Published:** 2021-02-15

**Authors:** Hiroaki Uchiyama, Yoshiki Nakamura, Seishirou Igarashi

**Affiliations:** Department of Chemistry and Materials Engineering, Kansai University 3-3-35 Yamate-cho Suita 564-8680 Japan h_uchi@kansai-u.ac.jp +81-6-6368-1121 extn 6131; Kansai University Japan

## Abstract

M-doped WO_3_ (M = Sn or In) films were prepared from aqueous coating solutions *via* evaporation-driven deposition during low-speed dip coating. Sn- and In-doping were easily achieved by controlling the chemical composition of simple coating solutions containing only metal salts and water. The crystallinity of the WO_3_, Sn-doped WO_3_, and In-doped WO_3_ films varied with heating temperature, where amorphous and crystalline films were obtained by heating at 200 and 500 °C, respectively. All the amorphous and crystalline films showed an electrochromic response, but good photoelectrochemical stability was observed only for the crystalline samples heated at 500 °C. The crystalline In–WO_3_ films exhibited a faster electrochromic color change than the WO_3_ or Sn–WO_3_ films, and good cycle stability for the electrochromic response in the visible wavelength region.

## Abstract 2

WO_3_ and M-doped WO_3_ (M = Sn or In) electrochromic films were obtained from aqueous solutions *via* evaporation-driven deposition. The In–WO_3_ films showed a faster electrochromic response than WO_3_ and Sn–WO_3_ films, and a good cycle stability.

## Graphical abstract

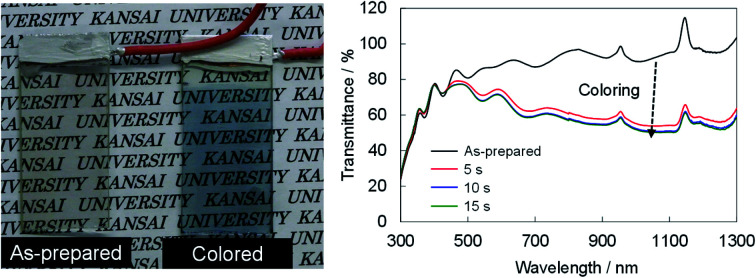

## Introduction

Electrochromic film materials are able to reversibly change their optical properties by applying an electrical voltage, and have thus raised much attention for various practical applications, such as in smart windows, display devices, and sensor materials.^1–5^ Tungsten oxide (WO_3_) is a typically used cathodic electrochromic material because of its relatively fast color change and good stability.^5–11^ Two-terminal electrochromic devices based on cathodic WO_3_ electrodes and appropriate anodic materials such as dimethyl ferrocene,^12^ hydroquinone,^13^ tetrathiafulvalene^14^ (soluble anodic species), and NiO,^15,16^ polyaniline^17^ (insoluble anodic species) have been widely studied for practical applications. The electrochromic reaction of WO_3_ can be represented as:WO_3_ + *x*M^+^ + *x*e^−^ ↔ M_*x*_WO_3_ (M^+^ = H^+^, Li^+^, or Na^+^)where the electrochemical insertion of cations (M^+^ = H^+^, Li^+^, or Na^+^) provides a reversible color change from transparent to blue.

The electrochromic performance of WO_3_ materials, including the response speed, the durability, and the degree of color change, is known to strongly depend on the crystallinity of the WO_3_ phase.^5,6,18–24^ The electrochromic behavior of amorphous WO_3_ is reported to be related to the polaron transition between W^4+^, W^5+^, and W^6+^ ions,^18,19^ and the coloration efficiency of amorphous materials is generally better than crystalline materials.^5,6,20–24^ However, amorphous WO_3_ films often dissolve in acidic electrolyte solutions, resulting in a low cycle stability.^5,6,20–24^ The electrochromism of crystalline WO_3_ can be discussed on the basis of the variation in electron density with enhanced electron scattering resulting from the intercalation of cations. Although crystalline WO_3_ films exhibit a high electrochemical stability and an optical modulation over a wide range of wavelengths, including the infrared region,^6^ the electrochromic response speed is relatively low.^5,6,20,21^ Therefore, controlling the crystallinity of the WO_3_ film material is essential for improving the electrochromic performance.

Doping with metal ions (such as Ni and Ti, among others) has been widely investigated as another strategy for making high-performance WO_3_ electrochromic materials.^24–27^ Bathe *et al.* investigated the influence of Ti doping on the electrochromic properties of WO_3_ thin films prepared by pulsed spray pyrolysis, and suggested that doping WO_3_ with Ti induced a phase transformation from monoclinic to amorphous and a rough surface morphology, resulting in improvement in the cycle stability, charge storage capacity, and reversibility of the films.^24^ Cai *et al.* prepared Ti-doped WO_3_ films with a hierarchical star-like structure by a hydrothermal method, where the star-like structure had a low charge-transfer resistance and ion diffusion resistance, leading to fast switching speed and high coloration efficiency.^26^ Zhou reported the preparation of Ni-doped WO_3_ films by a hydrothermal method, where the doping caused a distortion of the WO_3_ crystal structure and the formation of vertically aligned nanorods, which enhanced the optical modulation, coloration efficiency, and cycle stability.^27^ Such doping strategy has been also applied to other electrode materials than WO_3_, such as NiO. Kim *et al.* reported that the Cu doping into NiO films resulted in the high performance electrochromic supercapacitors.^28^ These results suggest that doping should allow us to achieve improvement in the electrochromic performance of WO_3_ materials.

**Fig. 1:**
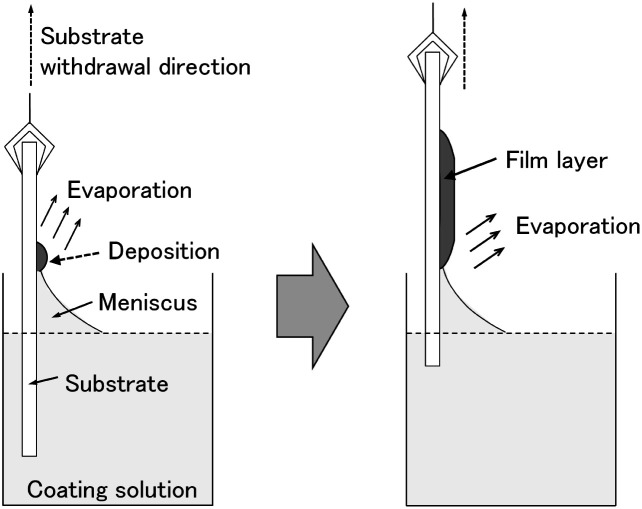
Schematic illustration of the evaporation-driven film deposition during low-speed dip coating.

In this work, we doped amorphous and crystalline WO_3_ films with tin (Sn^4+^) and indium (In^3+^) ions, and investigated the effect of the metal ion doping on the electrochromic properties of the WO_3_ films. The addition of Sn^4+^ or In^3+^ ions was expected to improve the durability of WO_3_ electrochromic films because these ions are electrochemically more stable than W^6+^ ions in aqueous media. Moreover, doping with low-valence metal ions should induce oxygen vacancies in the WO_3_ crystal lattice, which could influence the electron density in the WO_3_ phase, affecting the electrochromic performance. We recently suggested a low-speed dip-coating technique as a novel coating technique for making metal oxide thin films from organic-additive-free aqueous solutions.^29–31^Fig. 1 shows a schematic illustration of the film deposition during low-speed dip coating. The evaporation-driven deposition of solutes during dip coating with extremely low substrate withdrawal speeds would hinder the aqueous solution from gathering to form droplets, achieving a homogeneous deposition of a film layer on the substrate. The evaporation-driven deposition process does not need any organic additives for modifying the wettability of the aqueous coating solutions and, thus, pure inorganic precursor layers can be obtained on a glass substrate. Such inorganic precursors, thus obtained, would enable simple control of the crystallinity of WO_3_ films from amorphous to crystalline by varying the heating temperature. Moreover, the aqueous route is thought to allow for the doping of WO_3_ materials with various metal ions because water can dissolve many different metal salts. Here, we prepared amorphous and crystalline M-doped WO_3_ films (M = Sn or In) by a low-speed dip-coating technique with simple aqueous solutions containing only (NH_4_)_10_W_12_O_41_·5H_2_O, In(NO_3_)_3_·3H_2_O and SnCl_4_·5H_2_O, and evaluated their electrochromic properties. The effect of Sn- and In-doping on the electrochromic performance of WO_3_ films was studied by measuring the cyclic voltammogram and optical modulation during electrochromic reactions.

## Experimental

### Preparation of M-doped WO_3_ (M = Sn or In) thin films by low-speed dip coating

HCl aqueous solutions of pH 1.1–1.5 and NH_3_ aqueous solutions of pH 11.5 were prepared as the solvents for coating solutions by diluting with purified water *ca.* 36.0 mass% hydrochloric acid (Wako Pure Chemical Industries, Osaka, Japan) and *ca.* 10 mass% ammonia solutions (Wako Pure Chemical Industries, Osaka, Japan), respectively. Table 1 shows the compositions of the coating solutions for WO_3_ and M-doped WO_3_ (M = Sn or In) films.

**Table 1:** Compositions of the coating solutions, and thickness and M/W (M = Sn or In) mole ratio of the heat-treated films (500 °C, 0.5 h)

Film	Coating solutions				Heat-treated films	
	pH of solvents	[(NH_4_)_10_W_12_O_41_·5H_2_O]/mM	[SnCl_4_·5H_2_O]/mM	[InCl_3_·4H_2_O]/mM	Film thickness/nm	M (M = Sn or In)/W mole ratio^a^
WO_3_	1.5 (HCl)	10	—	—	34	—
Sn–WO_3_	11.5 (NH_3_)	5.0	6.0	—	35	0.085
In–WO_3_	1.1 (HCl)	5.0	—	6.0	38	0.12

a The mole ratio was analyzed by X-ray photoelectron spectroscopy (XPS).

1.25 g of (NH_4_)_10_W_12_O_41_·5H_2_O (Wako Pure Chemical Industries, Osaka, Japan) was added and dissolved in 40 cm^3^ of the HCl solutions of pH 1.5 under stirring at 60 °C. After stirring at 60 °C for 1 h, the resultant solutions served as WO_3_ coating solutions ([(NH_4_)_10_W_12_O_41_·5H_2_O] = 10.0 mM).

0.625 g of (NH_4_)_10_W_12_O_41_·5H_2_O and 0.0841 g of SnCl_4_·5H_2_O (Wako Pure Chemical Industries, Osaka, Japan) were added and dissolved in 40 cm^3^ of the NH_3_ solutions of pH 11.5 under stirring at 60 °C. After stirring at 60 °C for 1 h, the solutions served as Sn-doped WO_3_ (Sn–WO_3_) coating solutions ([(NH_4_)_10_W_12_O_41_·5H_2_O] = 5.00 mM, [SnCl_4_·5H_2_O] = 6.00 mM, and the Sn/W mole ratio was 0.1).

0.625 g of (NH_4_)_10_W_12_O_41_·5H_2_O and 0.0704 g of In(NO_3_)_3_·4H_2_O (Wako Pure Chemical Industries, Osaka, Japan) were added and dissolved in 40 cm^3^ of the HCl solutions of pH 1.1 under stirring at 60 °C. After stirring at 60 °C for 1 h, the solutions served as In-doped WO_3_ (In–WO_3_) coating solutions ([(NH_4_)_10_W_12_O_41_·5H_2_O] = 5.00 mM, [In(NO_3_)_3_·4H_2_O] = 6.00 mM, and the In/W mole ratio was 0.1).

WO_3_ and M-doped WO_3_ (M = Sn or In) precursor films were deposited on fluorine-doped tin oxide (FTO) glass substrates (20 mm × 40 mm × 1.0 mm) by a low-speed dip-coating technique. Low-speed dip coating was performed using a dip-coater (Portable Dip Coater DT-0001, SDI, Kyoto, Japan) in a thermostatic oven, where the substrates were withdrawn at 0.05 cm min^−1^. The coating temperature, *i.e.*, the temperature of the substrates, solutions, and atmosphere, was kept at 25 °C (for In-doped WO_3_ films) or 40 °C (for WO_3_ and Sn-doped WO_3_ films), where the solutions and substrates were heated at the prescribed temperature for 10 min in the thermostatic oven before the dip coating. The precursor films were heated in air at 200 °C for 24 h or at 500 °C for 0.5 h, where the precursor films were directly transferred to an electric furnace held at the prescribed temperature. Hereafter, the Sn- and In-doped WO_3_ films are denoted as Sn– and In–WO_3_ films.

### Characterization

Microscopic observation of the thin film samples was carried out using an optical microscope (KH-1300, HiROX, Tokyo, Japan). The microstructure of the thin films was observed using a field emission scanning electron microscope (FE-SEM; Model JSM-6500F, JEOL, Tokyo, Japan). The crystalline phases were identified using an X-ray diffractometer (Model Rint-Ultima III, Rigaku, Tokyo, Japan) with CuKα radiation operated at 40 kV and 40 mA at an incident angle of 0.5°. The chemical compositions of the product films were obtained using an X-ray photoelectron spectrometer (XPS; PHI5000 Versa Probe, ULVAC-PHI, Chigasaki, Japan) with a monochromatic AlKα X-ray source. The XPS analysis was done for the film samples coated on silica glass substrates, because FTO substrates containing Sn^4+^ ions inhibited the analysis of Sn–WO_3_ films. To counter the surface charging, a charge neutralizer was used during the collection of the spectra.

Film thickness was measured using a contact probe surface profilometer (SE-3500K31, Kosaka Laboratory, Tokyo, Japan). A part of the thin film was scraped off with a surgical knife immediately after the film deposition, and the level difference between the coated part and the scraped part was measured after heat treatment.

### Measurement of electrochromic properties

Electrochromic properties of the WO_3_ and M-doped WO_3_ films were evaluated at room temperature in a three-electrode cell using a potentiostat (HZ-7000, Hokuto Denko, Osaka, Japan) consisting of the film sample, a platinized Pt electrode, and a saturated calomel electrode (SCE) as the working, counter, and reference electrodes, respectively. An aqueous solution of 1 M H_2_SO_4_ was used as the supporting electrolyte.

Cyclic voltammetry (CV) was performed on the WO_3_ and M-doped WO_3_ films at a scan rate of 10 mV s^−1^ between −1.0 and 2.0 V *vs.* the SCE.

The optical modulation induced by electrochromic reactions was evaluated by an *in situ* optical absorption measurement. The three-electrode cell with circular silica windows (1.77 cm^2^) as a light path for an *in situ* UV-Vis-NIR absorption measurement was connected to a potentiostat, and set in the optical spectrometer (V-570, JASCO, Tokyo, Japan). The coloring and bleaching of the WO_3_ and M-doped WO_3_ films was carried out at −0.75 and 1.5 V *vs.* the SCE, respectively. The application of voltage was stopped at 5- (coloring) or 100- (bleaching) second intervals, and then optical absorption spectra were measured at wavelengths of 300–1300 nm. An FTO glass substrate was used as the reference for the optical measurement. Coloring and bleaching times were defined as the times when the variation in the optical absorption spectra stopped moving upon application of a voltage.

The cycle stability was tested by repeating the electrochromic color change. The coloring and bleaching cycles were repeated 50 times, where the coloring and bleaching of the films were performed by a voltage application of −0.75 V *vs.* the SCE for 20 s and 1.5 V *vs.* the SCE for 200 s, respectively. After 1 and 50 cycles, the optical absorption spectra of the film samples were measured.

## Results and discussion

### Preparation and characterization of WO_3_ and M-doped WO_3_ (M = Sn or In) films

**Fig. 2:**
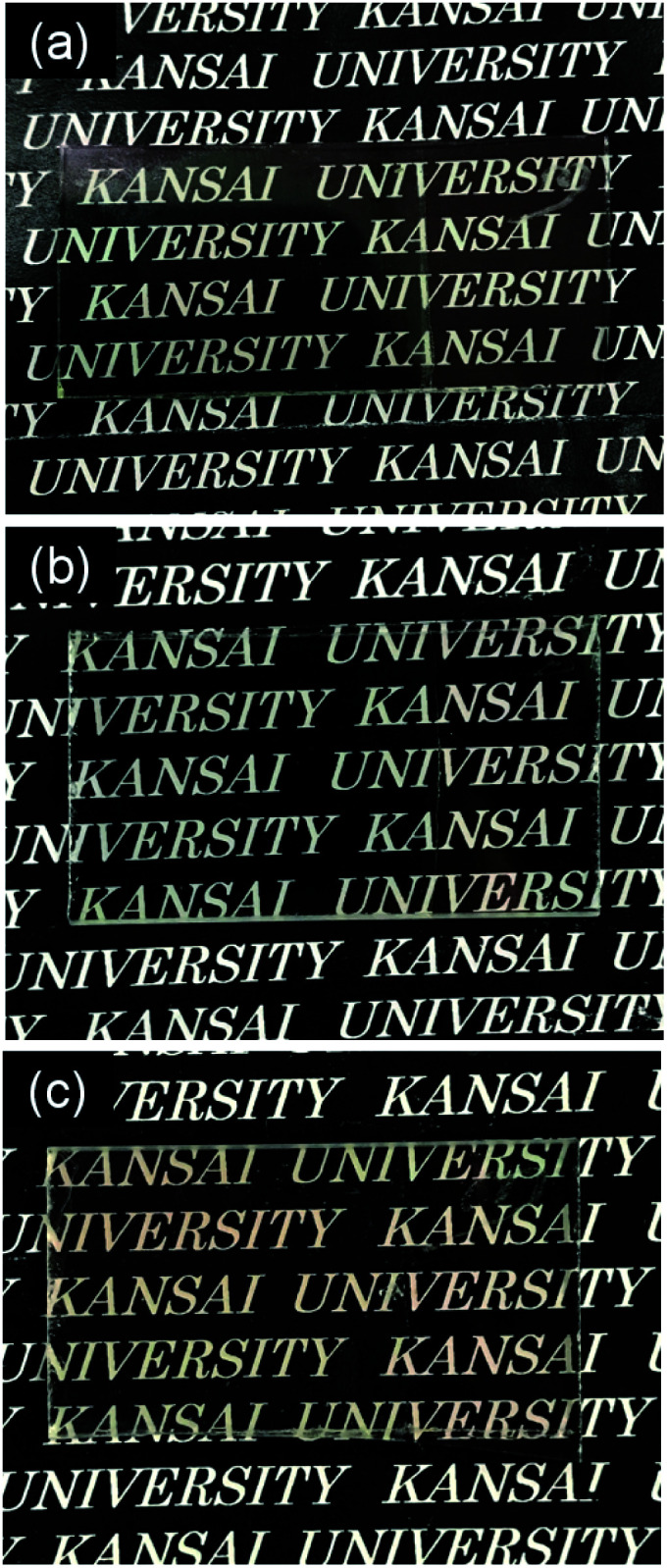
Optical micrographs of the WO_3_ (a), Sn–WO_3_ (b), and In–WO_3_ (c) films heated at 500 °C.

Coating solutions for the WO_3_ and In–WO_3_ films were prepared with HCl solutions of pH 1.5 and 1.1, respectively. However, coating solutions containing SnCl_4_ became cloudy under acidic conditions because of precipitate formation. Transparent coating solutions for Sn–WO_3_ films were obtained under alkaline conditions with NH_3_ solutions of pH 11.5. Precursor films were deposited on FTO glass substrates by low-speed dip coating, and then heated at 200 °C for 24 h or at 500 °C for 0.5 h for the thermal conversion to WO_3_, Sn–WO_3_, and In–WO_3_ films. Fig. 2 shows optical micrographs of the WO_3_, Sn–WO_3_, and In–WO_3_ films heated at 500 °C. All the product films were free from cracks and had a high level of transparency. In this work, we prepared Sn–WO_3_ and In–WO_3_ films with a wide range of M/W mole ratios (M = Sn or In) between 0.050 and 0.20. However, the Sn–WO_3_ films became cloudy when the Sn/W mole ratio was over 0.10, which may be because of the generation of SnO_2_ phase in the films. Therefore, the M/W mole ratio was fixed at 0.10 and these results are discussed hereafter. The thickness and the M/W (M = Sn or In) mole ratio of the product films heated at 500 °C are shown in Table 1. The film thickness was *ca.* 35 nm irrespective of whether doping was with Sn^4+^ or In^3+^ ions. The M/W mole ratios (M = Sn or In) in the Sn–WO_3_ and In–WO_3_ films, as evaluated by XPS, were 0.085 and 0.12, respectively, which closely agree with the compositions of the coating solutions (0.10) (the XPS spectra are shown in ESI Fig. S1†). In this work, low-concentration (NH_4_)_10_W_12_O_41_ aqueous solutions were used as the coating solutions ([(NH_4_)_10_W_12_O_41_·5H_2_O] = 5.00 or 10.0 mM) because tungsten salts are poorly soluble in many different solvents containing water and alcohols. However, homogeneous coating on the whole substrates was achieved with the low-concentration solutions *via* one-time coating. In the case of low-speed dip coating, the solvent preferentially evaporates at the edge of the meniscus, and the coating solutions are then locally concentrated there, resulting in the deposition of solutes on the substrates (Fig. 1).^29–31^ The evaporation-induced concentration enabled us to make homogeneous WO_3_ coating layers even from low-concentration solutions. Moreover, in this process, Sn- or In-doping was easily succeeded by the addition of tin or indium salts into the coating solutions.

**Fig. 3:**
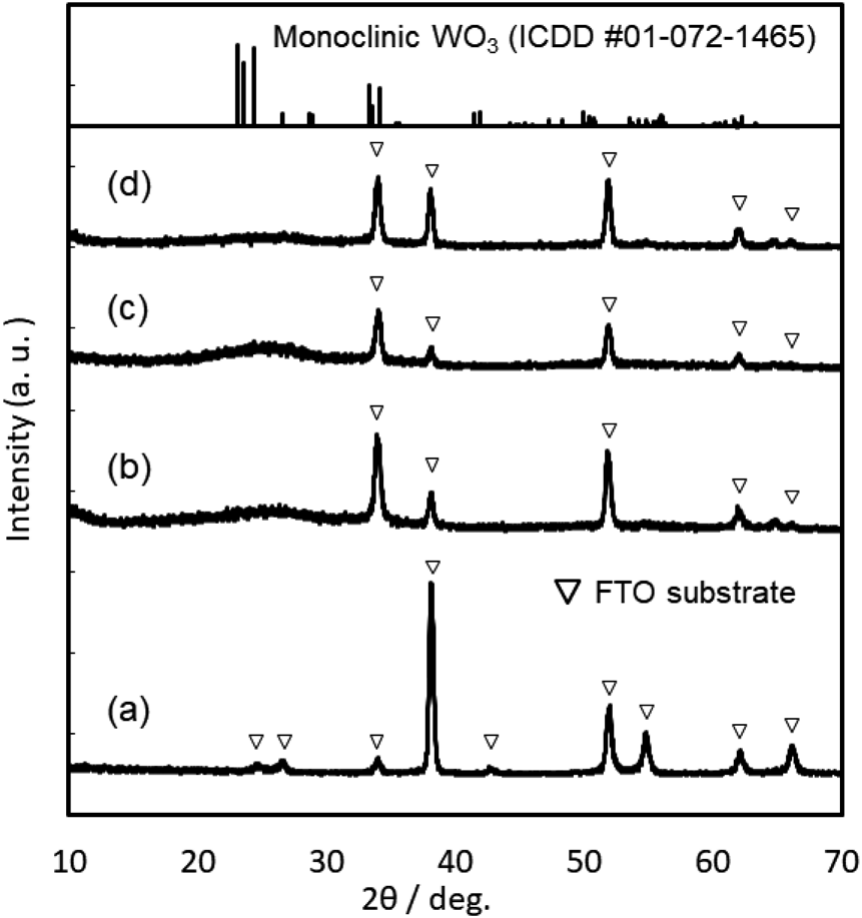
XRD patterns of the FTO substrate (a), and the WO_3_ (b), Sn–WO_3_ (c), and In–WO_3_ (d) films heated at 200 °C.

**Fig. 4:**
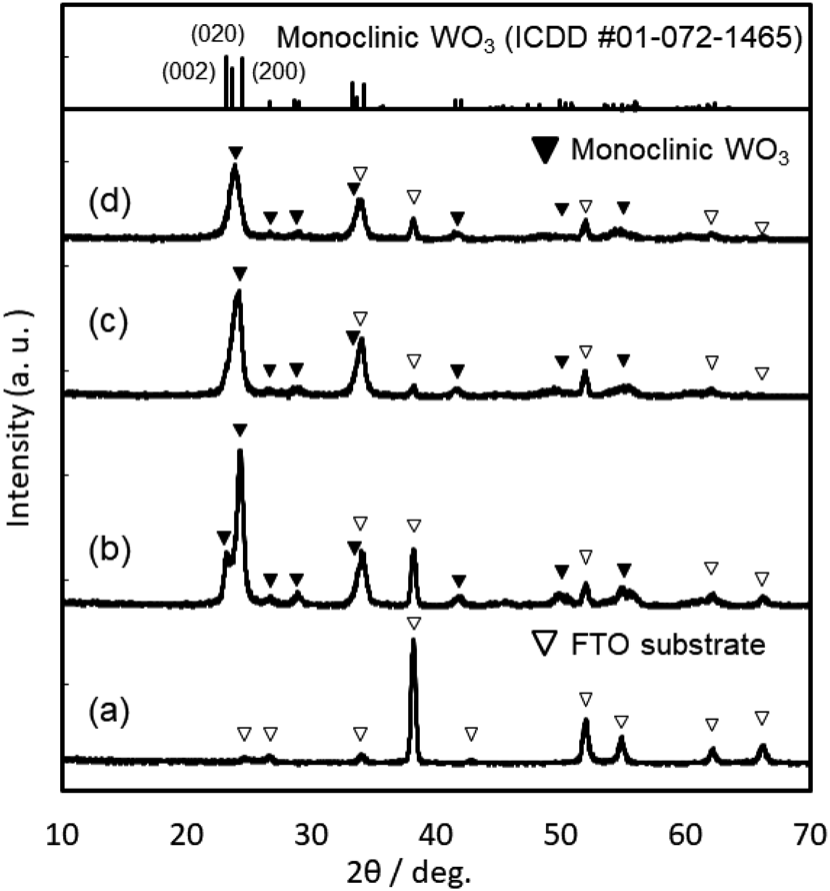
XRD patterns of the FTO substrate (a), and the WO_3_ (b), Sn–WO_3_ (c), and In–WO_3_ (d) films heated at 500 °C.

XRD patterns of the WO_3_, Sn–WO_3_, and In–WO_3_ films heated at 200 and 500 °C are shown in Fig. 3 and 4, respectively. Any diffraction peaks other than those due to FTO substrates were not observed for the WO_3_, Sn–WO_3_, or In–WO_3_ films heated at 200 °C (Fig. 3), which indicates that the films heated at this temperature consisted of an amorphous phase. Diffraction patterns attributable to the monoclinic WO_3_ phase appeared for all the films heated at 500 °C (Fig. 4), where SnO_2_ or In_2_O_3_ phases were not detected in the Sn–WO_3_ or In–WO_3_ films, respectively. The peak shift due to the substitution of Sn^4+^ or In^3+^ ions for W^6+^ ions was not clearly observed in the XRD patterns of the Sn–WO_3_ or In–WO_3_ films, while the diffraction peaks of the (002), (020), and (200) planes of monoclinic WO_3_ weakened and broadened upon the addition of Sn^4+^ and In^3+^ ions, which suggests that Sn- or In-doping could result in deformation of the WO_3_ lattice.

### Electrochromic properties of WO_3_ and M-doped WO_3_ (M = Sn or In) films

The electrochromic properties were evaluated for the amorphous and crystalline WO_3_, Sn–WO_3_, and In–WO_3_ films heated at 200 and 500 °C, respectively, where the electrochemical measurements were performed in an aqueous electrolyte of 1 M H_2_SO_4_. In this case, the coloring and bleaching of WO_3_ films progressed with the intercalation and deintercalation of H^+^ ions. The coloring of WO_3_ films is the following reduction reaction:^5^WO_3_ + H^+^ + e^−^ → HWO_3_ (deep blue color).

The bleaching of the films then follows the oxidation reaction:HWO_3_ → WO_3_ + H^+^ + e^−^ (light yellow color).

**Fig. 5:**
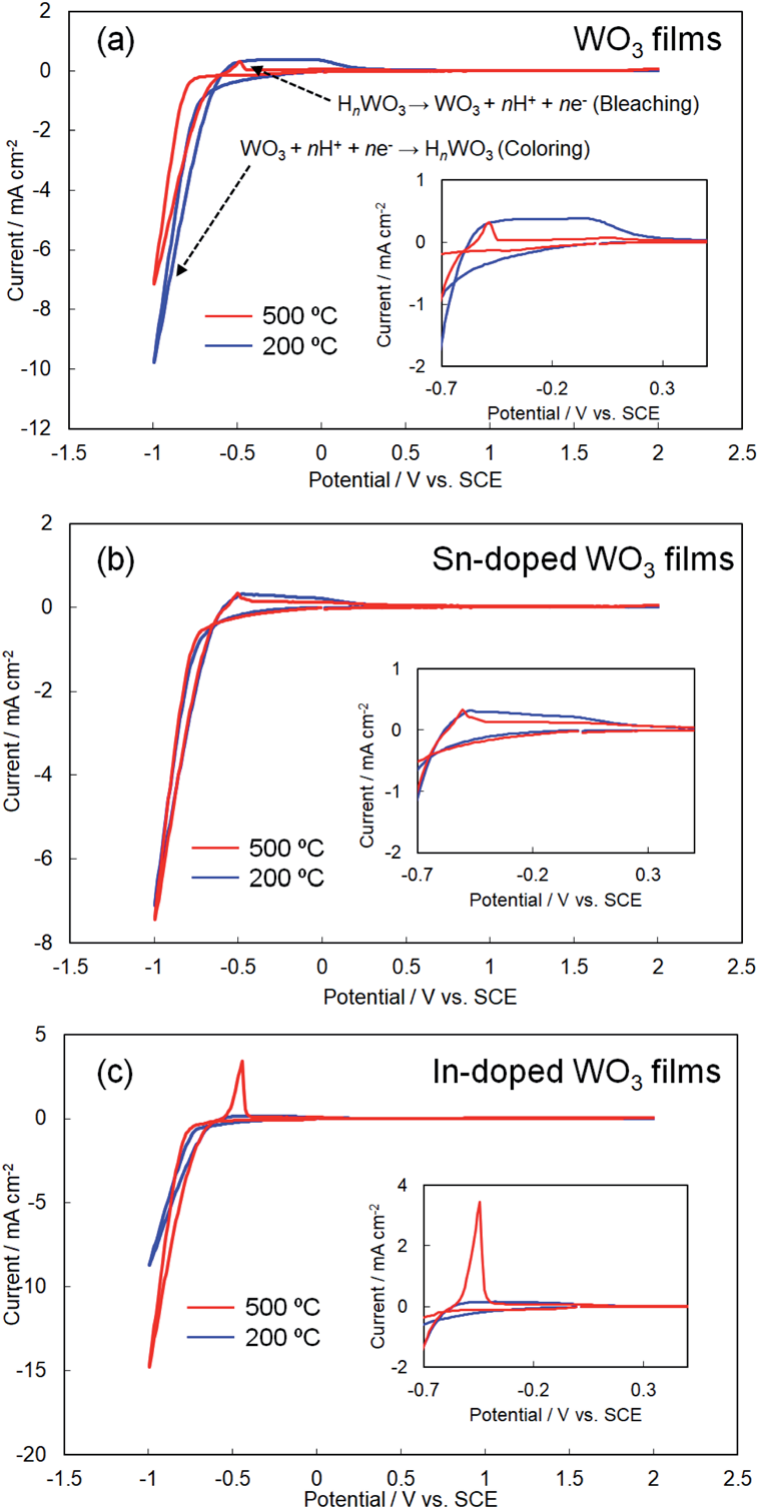
Cyclic voltammograms of the WO_3_ (a), Sn–WO_3_ (b), and In–WO_3_ (c) films heated at 200 and 500 °C.

**Fig. 6:**
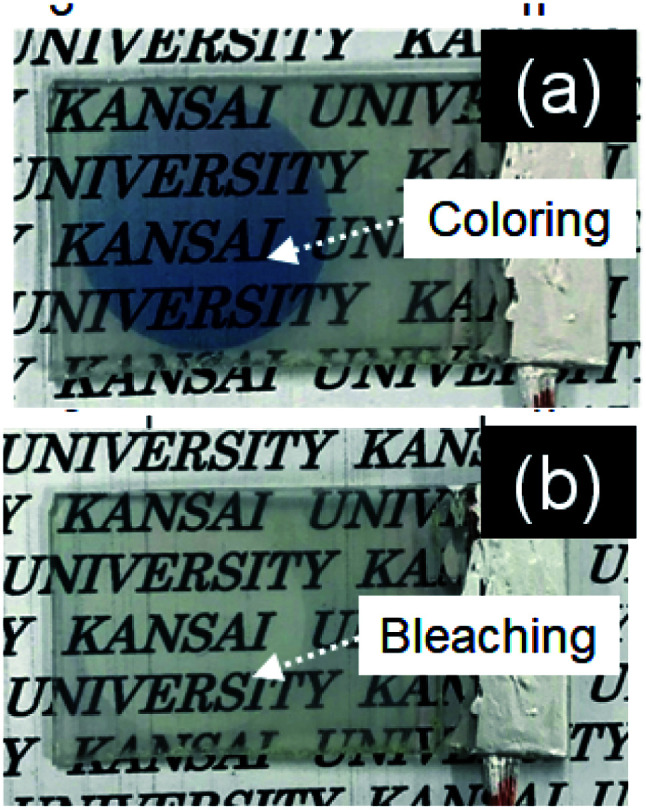
Optical micrographs of the colored (a) and bleached (b) WO_3_ films heated at 500 °C.

**Fig. 7:**
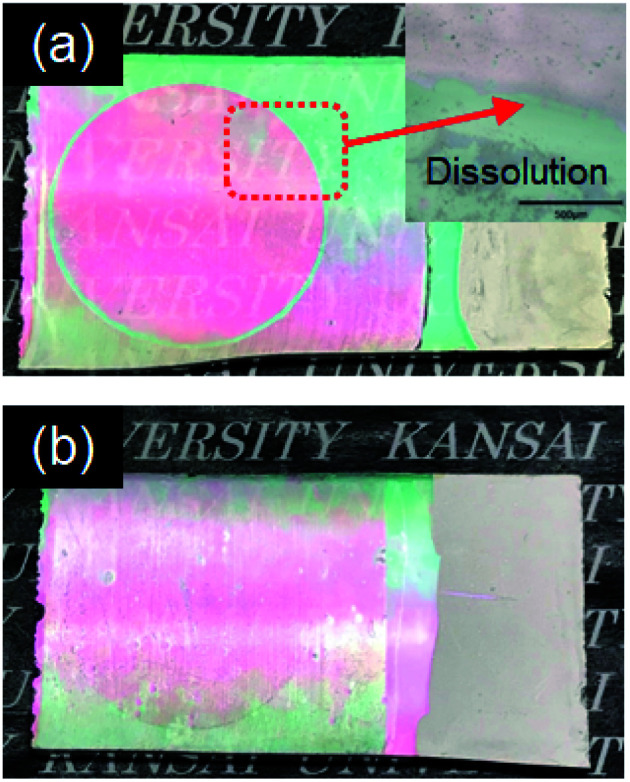
Optical micrographs of the amorphous (a) and crystalline (b) In–WO_3_ films after the coloring and bleaching cycle.

Fig. 5 shows cyclic voltammograms obtained for the amorphous and crystalline product films. Cathodic peaks from the reduction reaction with H^+^ ions were detected between −0.65 and −1.0 V *vs.* the SCE, irrespective of the heating temperature or the metal ion dopant used (Fig. 5), where the coloring of the films to deep blue was visually confirmed for all the films (see Fig. 6a). The cathodic response was almost unchanged with the variation in the crystallinity and the addition of Sn^4+^ or In^3+^ ions (Fig. 5). The anodic response due to the oxidation reaction was observed between −0.50 and 0.30 V *vs.* the SCE in the CV curves for all the films, and the bleaching of the blue color started there (Fig. 6b). The anodic peaks in the CV curves broadened with a decrease in heating temperature (Fig. 5), and the amorphous WO_3_, Sn–WO_3_, and In–WO_3_ films heated at 200 °C were partially dissolved after the coloring and bleaching cycle (Fig. 7a). Such dissolution of the films was not observed for the crystalline films heated at 500 °C (Fig. 7b). These results could indicate that the electrochemical stability of the amorphous films was relatively low, and the Sn- and In-doping did not improve the durability of the amorphous WO_3_ films. Therefore, hereafter, we mainly focused on the effect of the Sn- and In-doping on the electrochromic response of the crystalline WO_3_, Sn–WO_3_, and In–WO_3_ films heated at 500 °C.

**Fig. 8:**
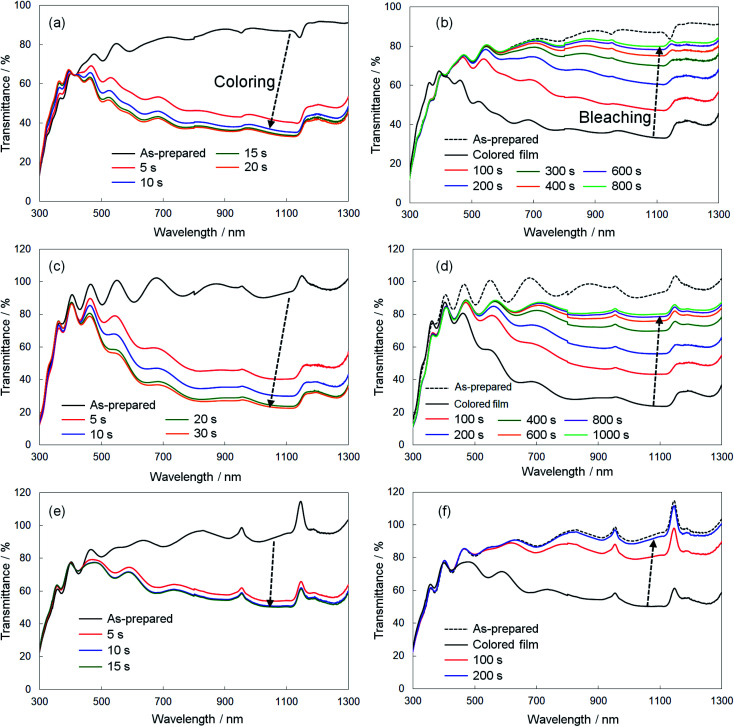
Variation in the UV-Vis-NIR absorption spectra of the crystalline WO_3_ (a and b), Sn–WO_3_ (c and d), and In–WO_3_ (e and f) films by voltage application. Coloring (a, c and e) and bleaching (b, d and f).

We evaluated the response speed of the electrochromic optical modulation for the crystalline WO_3_, Sn–WO_3_, and In–WO_3_ films, where the UV-Vis-NIR absorption spectra were measured when a voltage was applied. The coloring and bleaching of the films were carried out at −0.75 and 1.5 V *vs.* the SCE, respectively. Fig. 8 shows the variation in the UV-Vis-NIR absorption spectra of the crystalline WO_3_ (Fig. 8a and b), Sn–WO_3_ (Fig. 8c and d), and In–WO_3_ (Fig. 8e and f) films upon application of a voltage, and Table 2 shows the reaction time that was needed to finish the coloring and breaching of the films. The transmittance of the as-prepared crystalline WO_3_, Sn–WO_3_, and In–WO_3_ films was over 80% at wavelengths of 500–1300 nm (Fig. 8). The transmittance of the films decreased with the coloring by applying −0.75 V *vs.* the SCE. The coloring of the films concluded within 20 s for all the crystalline films, where the transmittance of WO_3_, Sn–WO_3_, and In–WO_3_ films at wavelengths greater than 600 nm was reduced to *ca.* 30, 20, and 40%, respectively (Fig. 8a, c, e and Table 2). The bleaching of the films was achieved by applying 1.5 V *vs.* the SCE. The crystalline WO_3_ and Sn–WO_3_ films needed around 800 s for bleaching, where the transmittance was not completely returned to that of the as-prepared state (Fig. 8b, d and Table 2). However, the blue color of the In–WO_3_ films rapidly bleached within 200 s, and the transparency almost completely returned to the as-prepared state (Fig. 8f and Table 2). Although the initial transmittance of colored In–WO_3_ films was relatively high (*ca.* 40%) (Fig. 8e), the response of the In–WO_3_ films was obviously faster than those of the WO_3_ and Sn–WO_3_ films. To our knowledge, the improvement in the electrochromic properties of WO_3_ materials by doping with In^3+^ ions has not been reported previously.

**Table 2:** Coloring and breaching time of the crystalline WO_3_, Sn–WO_3_, and In–WO_3_ films heated at 500 °C

Film	Coloring time/s	Bleaching time/s
WO_3_	15	800
Sn–WO_3_	20	800
In–WO_3_	10	200

The response speed of electrochromic materials is influenced by several factors, such as the porosity, crystallinity, and electron conductivity, among other factors. Because electrochemical reactions occur on the surface of electrode materials, the microstructures of the WO_3_ films can affect the electrochromic properties. However, there were no significant differences in the surface structures, as observed by FE-SEM, between the WO_3_, Sn–WO_3_, and In–WO_3_ films (ESI Fig. S2†). Disorder in the WO_3_ crystal lattice is also known to improve the electrochromic response because the resulting broadened channels can allow for smoother insertion of cations (such as H^+^ and Li^+^). Many researchers have reported that metal ion doping deformed the monoclinic structure of WO_3_ materials, providing faster electrochromic reactions.^24,26,27^ In the present work, Sn- and In-doping caused deformation of the WO_3_ lattice (Fig. 4). Thus, the improvement of response speed by In-doping was thought to be influenced by the deformation of the WO_3_ lattice (Fig. 4). However, the Sn-doping did not provide such a fast electrochromic response (Fig. 8c, d and Table 2), despite the lattice deformation. The difference in the effect on the electrochromic properties between the Sn- and In-doped materials might be caused by the difference in valence of the Sn^4+^*versus* In^3+^ ions. For crystalline WO_3_ materials, electrochromism is discussed on the basis of the variation in electron density with enhanced electron scattering from the intercalation of cations.^5^ The addition of In^3+^ ions might provide more oxygen vacancies than would Sn^4+^ ions, affecting the electrochromic behavior. To investigate the effect of the dopants, we attempted to investigate the valence states of W, In, and Sn elements in colored and bleached films by XPS. However, the thickness of the Sn–WO_3_ and In–WO_3_ films was very thin (*ca.* 35 nm) and the Sn and In contents were not sufficiently high (the Sn or In/W mole ratios were *ca.* 0.1). Thus, the surface impurities and the FTO substrates containing Sn^4+^ ions inhibited the precise analysis of the In and Sn elements, and consequently the difference in the valence states could not be adequately evaluated here. Detailed analysis of the valence states of Sn–WO_3_ and In–WO_3_ films remains a challenge.

**Fig. 9:**
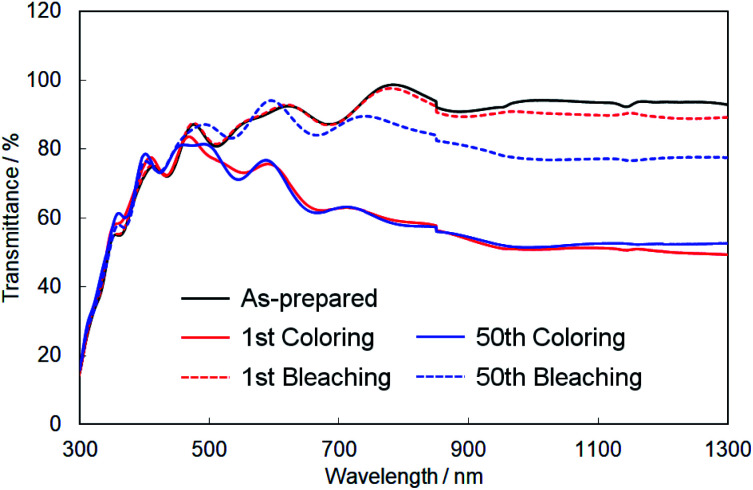
UV-Vis-NIR absorption spectra of the crystalline In–WO_3_ films after 1 and 50 electrochromic cycles.

Finally, we evaluated the cycle stability of crystalline In–WO_3_ films by repeating the coloring and bleaching. Fig. 9 shows UV-Vis-NIR absorption spectra of the crystalline In–WO_3_ films after 1 and 50 electrochromic cycles. The degree of coloring was maintained in the visible and near-infrared regions even after 50 cycles (Fig. 9). On the other hand, the transparency of the bleached states did not completely return to the as-prepared state in the near-infrared range over 800 nm after 50 cycles (Fig. 9). However, the bleached films after 50 cycles exhibited sufficiently high transmittance (over 80%) in the visible wavelength range between 400 and 800 nm (Fig. 9). These results show that the crystalline In–WO_3_ films have good cycle stability for use in electrochromic devices working at visible wavelengths.

## Conclusions

WO_3_ and M-doped WO_3_ (M = Sn or In) electrochromic films were obtained from aqueous solutions containing (NH_4_)_10_W_12_O_41_·5H_2_O, In(NO_3_)_3_·3H_2_O and SnCl_4_·5H_2_O by a low-speed dip-coating technique. Evaporation-driven deposition during low-speed dip coating enabled us to make homogeneous coating layers, even from low-concentration aqueous solutions. The Sn- and In-doping were easily achieved by controlling the chemical compositions of the solutions. The crystallinity of the WO_3_, Sn–WO_3_, and In–WO_3_ films was controlled by varying the heating temperature. The crystalline films heated at 500 °C showed high electrochemical stability, while the amorphous films obtained by heating at 200 °C dissolved after the electrochromic reactions. The crystalline In–WO_3_ films exhibited a faster coloring and bleaching response than did the WO_3_ and Sn–WO_3_ films, and this may be because of lattice deformation of the monoclinic WO_3_ phase and the change in valence states of the elements resulting from the In-doping. The crystalline In–WO_3_ films showed good cycle stability at visible wavelengths and are thus expected to be able to be applied to electrochromic devices.

## Conflicts of interest

There are no conflicts to declare.

## Supplementary Material

RA-011-D1RA00125F-s001
