# Supplementary material for: Aqueous synthesis of tin- and indium-doped WO3 films via evaporation-driven deposition and their electrochromic properties
Source: RSC Adv. 2021 Feb 15;11(13):7442–9. doi: 10.1039/d1ra00125f (PMC8695016; doi:10.1039/d1ra00125f)
Supplement: RA-011-D1RA00125F-s001 [file RA-011-D1RA00125F-s001.pdf]

Electronic Supplementary Material (ESI) for RSC Advances.
This journal is © The Royal Society of Chemistry 2021

# Aqueous synthesis of tin- and indium-doped $WO_3$ films via evaporation-driven deposition and their electrochromic properties

Hiroaki Uchiyama*, Yoshiki Nakamura and Seishirou Igarashi

E-mail address: h_uchi@kansai-u.ac.jp (H. Uchiyama).

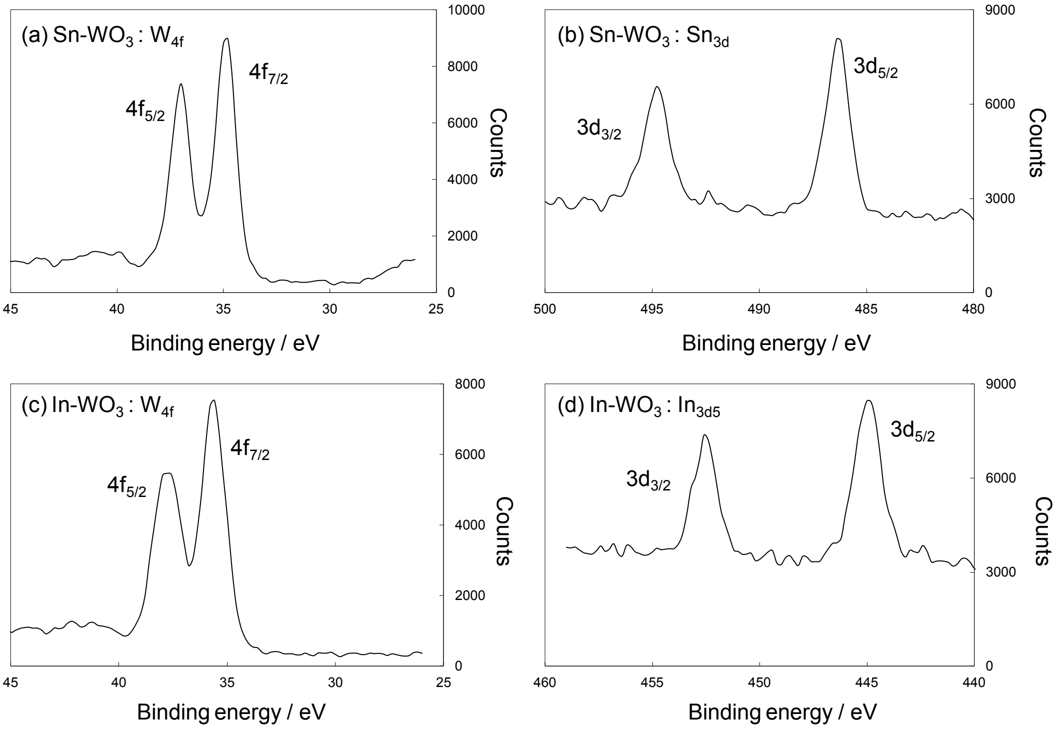


**Fig. S1** XPS spectra of the crystalline Sn-$WO_3$ (a: $W_{4f}$, b: $Sn_{3d}$) and In-$WO_3$ (c: $W_{4f}$, d: $In_{3d5}$) films heated at 500 °C.

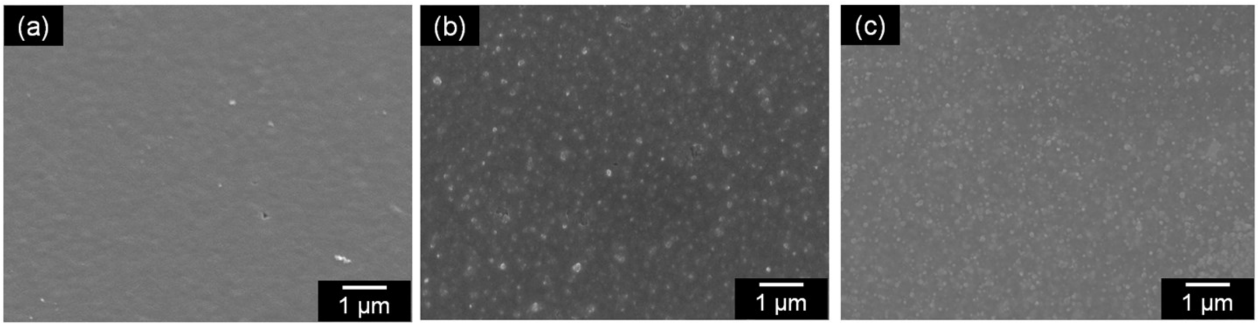


**Fig. S2** SEM images of the crystalline $WO_3$ (a), Sn-$WO_3$ (b), and In-$WO_3$ (c) films heated at 500 °C.
